# Penile Glans Necrosis following Prostatic Artery Embolization for the Treatment of Benign Prostatic Hyperplasia: A Rare but Serious Complication

**DOI:** 10.1155/2021/6662899

**Published:** 2021-03-13

**Authors:** Eric Chung

**Affiliations:** ^1^AndroUrology Centre, Brisbane QLD, Australia; ^2^University of Queensland, Princess Alexandra Hospital, Brisbane, QLD, Australia; ^3^Macquarie University Hospital, Sydney, NSW, Australia

## Abstract

**Background:**

Prostate artery embolization (PAE) is a novel endovascular procedure to treat men with benign prostatic hyperplasia (BPH) symptoms who wish to maintain sexual potency postoperatively. However, serious treatment-related adverse events (TRAEs) of PAE such as penile glans necrosis (PGN) can be devastating and require urgent attention. *Case presentation*. Mr GM is a 65-year-old sexually active Anglo-Saxon man who have long-standing BPH symptoms unresponsive to medical therapy. While he had an uneventful bilateral superselective PAE using gel foam, there were signs to suggest of PGN, and this was treated conservatively. The patient presented to the emergency department 5 days later with a painful, dark penile glans and accompanying urinary dysuria and hematuria. Clinical examination confirmed evolving PGN. He received 10 courses of hyperbaric oxygen therapy (HBOT) with complete resolution of his PGN.

**Conclusion:**

While superselective embolization is usually always performed, nontarget embolization may occur, as intravascular particles may reflux into adjacent vessels. In this unique and extremely rare case report of PGN following PAE, complete resolution was achieved with HBOT. Proposed benefits of HBOT include anti-inflammation, promotion of neovascularization, and induced rate of collagen deposition, resulting in a faster and more effective resolution of PGN.

## 1. Introduction

Prostatic artery embolization (PAE) is a relatively safe and effective treatment for men with lower urinary tract symptoms secondary to benign prostatic hyperplasia (BPH) [1, 2]. The mechanism of action for PAE to achieve targeted prostate tissue apoptosis occurs through catheter-directed particulate microembolic agents injected directly into the prostatic artery under fluoroscopic guidance [3, 4]. Given its unique and minimally invasive approach, PAE has gained considerable interest as an alternative to other contemporary minimally invasive BPH surgery that offers preservation of the sexual function postoperatively.

Common treatment-related adverse events (TRAE) include urinary tract infection, acute urinary retention, dysuria, and persistent urinary symptoms [1, 2]. However, serious TRAE can occur when nontarget embolization of intravascular particles refluxes into adjacent penile, vesical, or rectal arteries causing bladder wall ischemia, ischemic glans of the peni,s and ischemic rectitis [1, 5, 6]. We present a rare case of penile glans necrosis (PGN) secondary to nontarget embolization during PAE and the role of hyperbaric oxygen therapy (HBOT).

## 2. Case Report

This case report has received institutional ethics review board approval and patient informed consent. Mr GM is a 65-year-old sexually active man who has long-standing BPH symptoms. He has tried oral Tamsulosin therapy with minimal clinical efficacy and is bothered by the retrograde ejaculation. He has a normal erectile function and is sexually active. His other medical history includes hypertension, hyperlipidemia, and gout.

Following discussion regarding various minimally invasive BPH surgeries, he has elected to undergo PAE to improve his urinary flow while at the same time preserve his sexual function. Preoperative planning was undertaken using computer tomography angiography (CTA) pelvis which showed a prostatic volume of 138 cc (68 × 60 × 65 mm).

**Figure 1:**
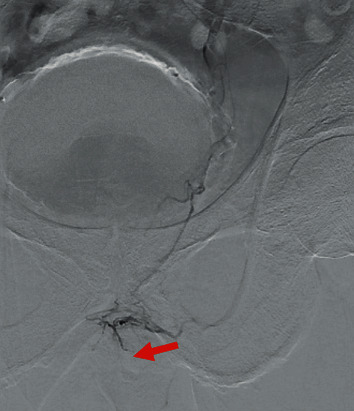
Presence of collateral vessels from the lateral prostatic artery to the penis and nontarget embolization of the penile artery (arrow).

A single operator, who had 15 years of experience in endovascular procedures and 5 years of experience in PAE, performed bilateral superselective PAE under local anesthesia with mild sedation. The total time taken for PAE was 118 minutes. Under ultrasound-guided left brachial artery puncture, a 4 French CXI guide catheter placed into the anterior division of the internal iliac arteries on both sides. Headway duo microcatheter was used for selective right prostate artery catheterization. This was uneventful with good uptake of embolic into the prostate with no observed collateral circulation. On the left access to the prostate artery was difficult. It came off the anterior division of the internal iliac artery with two other vessels, both of which went to the bladder. It was very difficult to select the prostate artery despite it being quite large. This was eventually catheterized using a synchrosoft guidewire with a very marked hook shape on the end. Flat-panel CT showed a satisfactory appearance to the preembolization picture with the only prostate selected. During the course of the embolization, it became clear that collateral vessels from the lateral prostatic artery to the penis had opened up (Figure 1). This was treated with Gelfoam with further Gelfoam used at the end of the embolization procedure. A reasonable volume of embolic injected on this site, and hemostasis achieved without difficulty.

Immediately postembolization, Mr GM complained of perineal discomfort and urinary frequency. These symptoms are thought to be secondary to some off-target embolization to the tip of the penis as the arteries to the penis opened up during the course of embolization of the left-sided prostate artery. The patient developed a small area of punctate purple discoloration at the tip of the penis with sensitivity within the distal urethra and hypersensitivity and a painful glans penis. Since the patient was able to void, he was discharged home with simple analgesia.

**Figure 2:**
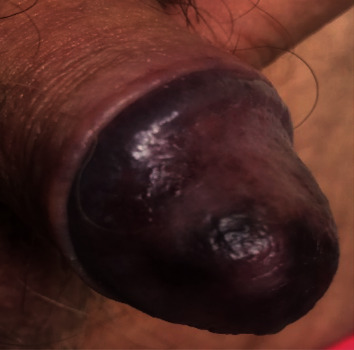
The appearance of penile glans necrosis at day 5 of postembolization.

However, the patient presented to the emergency department 5 days after embolization with a painful, dark glans penis consistent with an evolving PGN (Figure 2). He reports erectile dysfunction although he denied bowel issue, sensory change, or motor weakness to his gluteal or lower limb.

Following a discussion on his condition and various treatment options, he was referred to receive HBOT and completed 10 courses of therapy. At a follow-up visit 4 weeks following his last HBOT session, he reported resolution of his penile pain, glans discoloration, dysuria, and hematuria. The patient reported normal penile sensation, regained normal erection, and engaged in satisfactory sexual activity. Clinical examination showed almost complete resolution of his PGN.

## 3. Discussion

Preoperative workup includes CTA of the prostatic vessels to determine underlying prostatic vascular anatomy since multiple pelvic vessel anastomoses can present considerable technical challenges [4]. If significant vascular anastomoses are identified, the angiocatheter can be positioned distal to the vessel, or protective coil embolization is performed. Hence, PAE outcome can be variable and is highly operator-dependent; although there are concerted efforts to standardize and improve the reproducibility of PAE. Benign TRAE accounts for over 99% of all complications, and these include post-PAE syndrome, dysuria, urinary tract infection, hematuria, hematospermia, and urinary retention while serious complications due to nontarget embolization can result in ischemia of bladder, rectum, or penis [1–4]. However, the large variations in prostatic artery anatomy with collateral anastomoses can lead to nontarget embolization with subsequent ischemia, inflammation, and ulceration of affected organs such as PGN [6].

While the exact mechanism by which HBOT works is debatable, it is theorized that HBOT improves the oxygen concentration in a person's blood, thereby increasing the amount of oxygen reaching the areas that need to heal [7]. There are several beneficial properties of HBOT concomitant with the elevated oxygen distribution in tissue including anti-inflammation and promotion of neovascularization through vascular endothelial growth factor proliferation, augmented fibroblast, lymphocyte and macrophage activity, tissue, and wound repair as well as bactericidal activity. Furthermore, it is thought that HBOT may be able to support these mechanisms by increasing the amount of oxygen in the blood and directing it to the regions where it is needed most, thereby decreases the necessary time for wound healing as well as the induced rate of collagen deposition [7].

## 4. Conclusion

This unique but devastating case of PGN secondary to PAE illustrates the importance of discussing the risk of nontarget embolization with patients, and that HBOT can be an effective treatment option. As the practice of PAE takes off, education and mentoring are essential for its safe uptake worldwide. Clinicians must understand PAE's role with its strengths and weaknesses to enable optimal patient selection, ensure the best results, and minimize complications.

## Data Availability

Data is included in the manuscript.
